# Complete Genome Sequence of *Humibacter aquilariae* BT305, Isolated from the Small Intestine of Castrated Beef Cattle

**DOI:** 10.1128/MRA.01511-18

**Published:** 2019-01-24

**Authors:** Tae Woong Whon, Jin-Woo Bae

**Affiliations:** a Department of Biology and Department of Life and Nanopharmaceutical Sciences, Kyung Hee University, Seoul, Republic of Korea; Indiana University, Bloomington

## Abstract

Members of the genus Humibacter, from the family *Microbacteriaceae*, have been isolated from sewage sludge compost, agarwood chips, and various soil samples. Here, we report the complete genome sequence of Humibacter aquilariae BT305, the first genome sequence identified within the genus Humibacter, which was isolated from the small intestine of castrated beef cattle.

## ANNOUNCEMENT

The genus Humibacter was first proposed by Vaz-Moreira et al. (1), with Humibacter albus as the type species. The genus belongs to the family *Microbacteriaceae* in the phylum *Actinobacteria*, and Humibacter spp. have been isolated from a wide variety of natural sources, such as sewage sludge compost (1), agarwood chips (2), soil samples from natural caves (3), white heron nesting sites (4), and agricultural fields (5). Members of the genus Humibacter are aerobic, Gram positive, motile or nonmotile, and have a short rod-like shape. Their cells contain ornithine and 2,4-diaminobutyric acid (DAB) in the cell wall peptidoglycan, *N*-acetylated murein, and major menaquinones (MK) 11 and 12 (1–5). Genomic analyses of closely related genera (e.g., Leifsonia) belonging to the family *Microbacteriaceae* have revealed that these microbes can promote plant growth (6) and heavy metal resistance (7), suggesting that they may have agricultural applications.

Humibacter aquilariae BT305 was isolated from the small intestine of adult castrated beef cattle. The luminal content of the ileum was collected from a local slaughterhouse (Gunwi-Gun, South Korea). The study protocol was approved by the institutional review board of Kyung Hee University [KHUASP, (SE)-17-026], and the experiments were performed in agreement with the ARRIVE guidelines (8). The isolate was cultivated on brain heart infusion (BHI) agar (Becton, Dickinson, Franklin Lakes, NJ, USA) under aerobic conditions for 36 h at 30°C. Genomic DNA of the cultured isolate was extracted using the MG genomic DNA purification kit (MGmed, Seoul, South Korea), according to the manufacturer’s instructions. The whole-genome sequence of H. aquilariae BT305 was obtained by next-generation sequencing on the PacBio RS II (20-kb SMRTbell template) and Illumina HiSeq 4000 (TruSeq DNA PCR-free 350-bp library) platforms. To obtain the 20-kb library, genomic DNA was sheared with g-TUBE (Covaris) and purified using AMPure PB magnetic beads (Beckman Coulter). The sequencing library for Illumina HiSeq 4000 platform was prepared by random fragmentation of the DNA sample, followed by 5′ and 3′ adapter ligation. Quality control of the Illumina reads was performed to qualify reads with a Phred score of >30 for downstream analysis. The PacBio reads were quality filtered and *de novo* assembled using RS HGAP Assembly version 3.0 and polished with Quiver. The assembly of H. aquilariae BT305 was annotated using the RAST prokaryotic genome annotation server (http://rast.nmpdr.org/) (9). Prophage insert regions were searched using the online phage search tool PHASTER (10). RNAmmer 1.2 (11) and tRNAscan-SE 1.21 (12) were used to identify rRNA and tRNA sequences, respectively.

The filtered data generated a total of 2,074,712,267 bases and 6,872,355 reads in 2 contigs. The *N*_50_ value after assembly correction was 3,744,173 bp. The complete genome sequence of H. aquilariae strain BT305 consisted of a single circular chromosome of 3,744,173 bp, with a GC content of 70.8%. The sequencing coverage was 228-fold. The genome contained 3,568 coding sequences, 53 tRNAs, and 6 rRNAs. A linear plasmid of 121,511 bp, with 27-fold coverage, was identified along with the genome sequence. The plasmid contained 136 coding sequences with no tRNAs or rRNAs. RAST annotation revealed that the genome contained 3,592 coding sequences and 51 RNAs. The genome contained two incomplete prophages, named PHAGE_Bacill_G_NC_023719(2), and PHAGE_Achrom_JWAlpha_NC_023556(1), at bp positions 63747 to 73218 and 526181 to 534340, respectively.

### Data availability.

The complete genome data of Humibacter aquilariae BT305 were deposited under SRA BioProject number PRJNA482061, BioSample number SAMN09694957, and GenBank accession numbers CP031192 for the chromosome and CP031193 for the plasmid.
